# Essentials of Physics

**Published:** 1892-03

**Authors:** 


Essentials of Physics. Arranged in the form of Questions and Answers. Prepared especially for Students of Medicine. By Fred. J. Brockway, M. D., Assistant Demonstrator of Anatomy at College of Physicians and Surgeons, New York; with 155 illustrations. Philadelphia: W. B. Saunders, 913 Walnut Street, 1892. Price, $1.00, net.

Mr. Saunders is the publisher of an admirable series of Question-Compends of small octavo size, uniformly bound in blue cloth, and sold at a moderate price. The volume now under notice is No. 22 of the series.

In these days, so many publications calculated to assist the student in acquiring knowledge and fitting himself for examination are issued, that there can be but little excuse for his failing to learn the principles of his profession thoroughly. In the department of physics and chemistry the student cannot afford to be deficient. They constitute two of the foundation-stones of his medical knowledge. He should indeed have acquired them before he begins the study of medicine. But if he have not, or if he have become "rusty" in such knowledge, this admirable little book now before us will fulfill the need of a suitable instructor. The arrangement is excellent. Each paragraph is headed by a question in heavy black letter, and the answer is a copious and ably written explanation, illustrated by fine wood cuts. This little book goes upon the editorial shelf as a work of reference. W. M. J.

